# Interplay of Adsorption Geometry and Work Function Evolution at the TCNE/Cu(111) Interface

**DOI:** 10.1021/acs.jpcc.3c06422

**Published:** 2023-12-08

**Authors:** Max Niederreiter, Johannes Cartus, Anna Werkovits, Oliver T. Hofmann, Thomas Risse, Martin Sterrer

**Affiliations:** † Institute of Physics, University of Graz, NAWI Graz, Universitätsplatz 5, 8010 Graz, Austria; ‡ Institute of Solid State Physics, Graz University of Technology, NAWI Graz, Petersgasse, 16/II, 8010 Graz, Austria; § Institute of Chemistry and Biochemistry, Freie Universität Berlin, Arminallee 22, 14195 Berlin, Germany

## Abstract

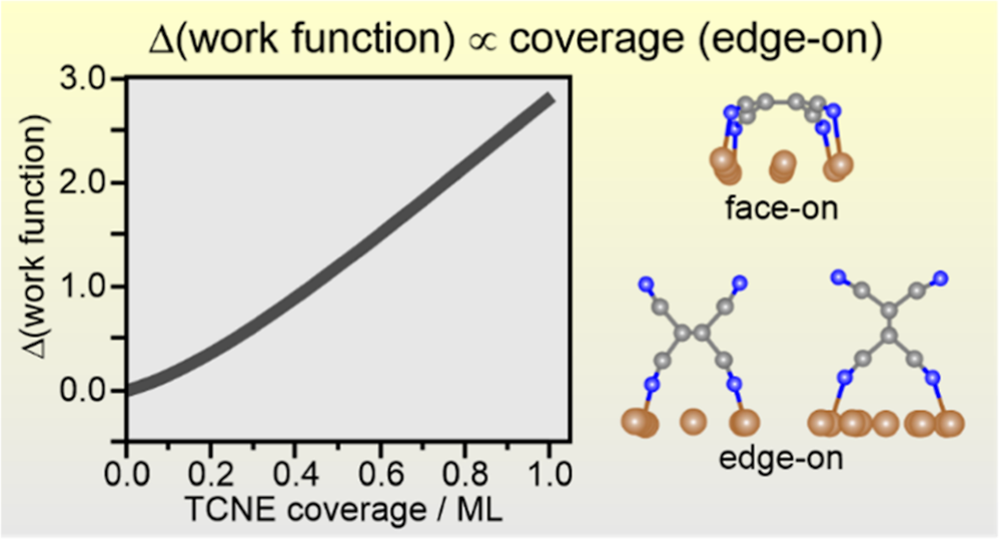

The adsorption of organic electron acceptors on metal surfaces is a powerful way to change the effective work function of the substrate through the formation of charge-transfer-induced dipoles. The work function of the interfaces is hence controlled by the redistribution of charges upon adsorption of the organic layer, which depends not only on the electron affinity of the organic material but also on the adsorption geometry. As shown in this work, the latter dependence controls the work function also in the case of adsorbate layers exhibiting a mixture of various adsorption geometries. Based on a combined experimental (core-level and infrared spectroscopy) and theoretical (density functional theory) study for tetracyanoethylene (TCNE) on Cu(111), we find that TCNE adsorbs in at least three different orientations, depending on TCNE coverage. At low coverage, flat lying TCNE dominates, as it possesses the highest adsorption energy. At a higher coverage, additionally, two different standing orientations are found. This is accompanied by a large increase in the work function of almost 3 eV at full monolayer coverage. Our results suggest that the large increase in work function is mainly due to the surface dipole of the free CN groups of the standing molecules and less dependent on the charge-transfer dipole of the differently oriented and charged molecules. This, in turn, opens new opportunities to control the work function of interfaces, e.g., by synthetic modification of the adsorbates, which may allow one to alter the adsorption geometries of the molecules as well as their contributions to the interface dipoles and, hence, the work function.

## 1 Introduction

The characteristics of organic electronic devices depend acutely on the properties of the interfaces between their individual constituents.^1−3^ One such interface property is the interface dipole established between a metallic contact and an organic semiconductor, which, intended or not, affects many other interface properties like energy level alignment and, hence, charge injection barriers.^2,4^ Understanding the formation of the interface dipole and manipulating it to suit specific applications are, therefore, principal goals in interface engineering.^5^

Several approaches have been pursued to control interface dipoles by organic layers using self-assembled monolayers of molecules exhibiting dipolar functional groups, which give rise to changes of the work function in case of properly aligned dipoles.^5−10^ Alternatively, the adsorption of strong electron donors or acceptors, which undergo charge transfer with the substrate, can be employed to modify the interface dipole. Empirically, the interface dipole of such charge-transfer adsorbate layers was often found to be (more or less) insensitive to the underlying substrate, resulting in a defined absolute work function independent of the nature of the underlying substrate.^1,11,12^ This phenomenon is known as “Fermi-level pinning”.^1,9,11^ Although there are different models as to which property of the organics the Fermi-level is pinned (polaronic state, integer charge-transfer states, molecular LUMO, etc.),^1,13−16^ there is consensus that better electron acceptors (i.e., larger electron affinities) generally lead to larger interface dipoles.^2^ In this regard, it is important to note that the adsorption geometry can strongly influence the interface dipole also for Fermi-level-pinned systems because the charge state of the adsorbate can depend on the adsorption geometry.^17−19^

**Figure 1:**
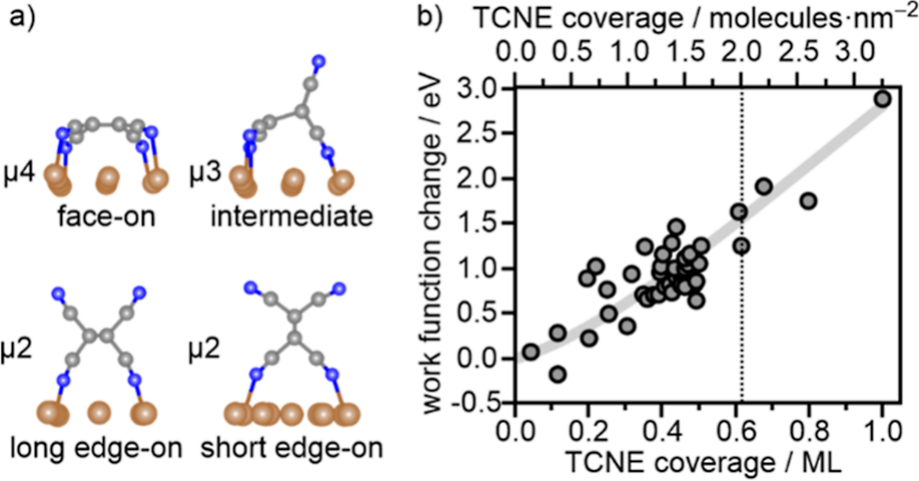
(a) Models of TCNE adsorption geometries on Cu(111). Adapted with permission from ref (19). Copyright 2020 Wiley-VCH, ref (30). Copyright 2022 American Chemical Society. (b) Experimentally determined change of the work function of Cu(111) with increasing TCNE coverage. The work function of the pristine surface Φ(θ = 0) = 4.7 eV. The light gray line is a guide to the eye to demonstrate the approximately linearly increasing trend of ΔΦ with TCNE coverage.

Tetracyanoethylene (TCNE) is a prototypical adsorbate in this respect, as it not only has a high electron affinity, which renders negatively charged molecules on most metal surfaces,^19−26^ but also contains four dipolar nitrile groups, whose orientations can impact the interface dipole and, thus, the work function.^27^ Various ordered structures of strongly bound TCNE molecules with face-on (flat-lying) adsorption geometry (μ4, all four CN groups bonded to the surface, Figure 1a) were found on Ag and Cu single-crystal surfaces.^23−26^ In both cases, there are strong experimental indications for charge transfer between the organic molecule and the surface. In contrast, the TCNE molecules in the ordered aggregates formed at room temperature on the inert Au(111) surface are uncharged and adsorb in an edge-on (upright) geometry with only two nitrile groups bonded to the surface (μ2, Figure 1a).^26,28^ A more recent study on the monolayer structure of TCNE on Co(100) suggested that although the face-on geometry (μ4) dominates in the initial stages of adsorption, molecules with the edge-on motif (μ2) appear at a higher coverage.^29^ The latter reorientation is particularly interesting as measurements of the work function showed indeed a maximum TCNE-induced work function increase of 1 eV for the mixed (μ4+μ2) monolayer.^29^ An even higher work function increase of up to 3 eV was predicted by some of us for a monolayer consisting of only edge-on TCNE on Cu(111).^27^ Additionally, a transition from stable face-on molecules at low coverage to edge-on molecules at higher coverages was put forward based on density functional theory (DFT) calculations^19,30^ as a more likely explanation for the results of early vibrational spectroscopy experiments for TCNE on Cu(111), which were originally interpreted as doubly charged molecules in the first and singly charged ones in the second layer.^21,22^

With respect to the expected effect of the coverage-dependent reorientation of the molecules on the work function, it is important to realize that such a transition from face-on molecules, which are more strongly bound, to edge-on molecules, which have a higher overall adsorption energy due to the smaller footprint that enables higher coverages of the adsorbed molecules, is a kinetically controlled process. This renders the formation of adsorbate layers with mixed orientations under the experimental conditions likely. In this respect, the important question arises if for layers with mixed orientations the interface dipole can be understood by an average of the contributions of the different surface dipoles, or if an alternative explanation is required?

To address this question, we investigated the adsorption of TCNE on Cu(111) at room temperature using spectroscopic methods to establish a connection between the work function of the system and the geometry of the adsorbed molecules. X-ray photoelectron spectroscopy (XPS) is used to distinguish between TCNE molecules with face-on and edge-on orientations via different charge transfer-dependent core-level shifts.^29^ Infrared reflection absorption spectroscopy (IRRAS) is sensitive to both the charge state of TCNE through the frequency of certain vibrational modes and also to the orientation of an adsorbate due to the metal surface selection rule and thus provides complementary insights to XPS as well as a point of comparison to previous studies. The experimental results are compared with computationally predicted work functions of mixed adsorbate layers.

## 2 Methods

### 2.1 Experimental Details

The experimental setup consists of an ultrahigh vacuum chamber equipped with a low-energy electron diffraction (LEED) optics, a dual-anode (Mg/Al) X-ray source (Specs XR50), a hemispherical electron analyzer (ScientaOmicron Argus CU), a mass spectrometer, a sputter gun, and a quartz-crystal microbalance. Attached to this chamber is a dedicated small chamber for IRRAS measurements, where the unit for TCNE dosing is installed. The Cu(111) sample was mounted on a flag-style sample plate with direct thermocouple (type K) connection to the sample and held by a manipulator. The sample was cooled with liquid nitrogen to 100 K and heated by electron beam heating.

The Cu(111) single crystal was cleaned by repeated cycles of Ar^+^-ion bombardment and annealing at 850 K until a clean and well-ordered surface was obtained, as was verified with LEED and XPS. TCNE was dosed from an evacuated glass vessel through a leak valve and the dose was controlled through pressure and exposure time. Prior to the dosing experiments, TCNE was resublimated to increase its purity. Except for experiments requiring multilayer coverage of TCNE, where the sample temperature had to be lowered to 200 K for dosing, all other experiments reported in this work were conducted with the sample held at room temperature.

The IRRAS experiments were performed with a Bruker Vertex 80v FTIR spectrometer and a mercury cadmium telluride detector. TCNE was directly dosed with the sample in the IRRAS measurement position, allowing the evolution of IR signals to be monitored in situ. The resolution was set to 4 cm^–1^ and between 100 and 500 scans were accumulated for one spectrum. XPS measurements were taken at normal emission with unmonochromated Al K_α_ excitation. Detail spectra recorded in the C 1s and N 1s binding energy region were used for determination of the coverage and the distribution of different adsorbed TCNE species, as described in more detail in the Supporting Information. The work function was obtained from measurements of the secondary electron cutoff in a sample bias configuration (Figure S1, Supporting Information).

### 2.2 Computational Details

All computational settings were reused from earlier works,^19^ where they have been carefully converged. For consistency, the important settings are repeated here. The DFT calculations were carried out with the FHI-aims code^31^ using the Perdew–Burke–Ernzerhof functional^32^ and a dispersion correction scheme (vdW^surf^).^33^ The calculations were done using a repeated slab approach, decoupling the periodic replica in z-direction using a dipole correction.^34^ The substrate slabs were modeled using 7 layers of Cu and were separated by at least 44 Å of vacuum. The SCF iterations were executed until the change in the total energy fell below 10^–5^ eV and the change in the electron density fell below 10^–2^ electrons/Bohr^3^ between subsequent iterations. We used a Gaussian occupation scheme with a broadening of 0.1 eV together with a generalized Monkhorst–Pack *k*-point grid^35,36^ with a maximal spacing of Δ*k* = 2π/80 Å^–1^. Furthermore, we used a mixed basis set based on the “tight” species defaults of FHI-aims for the uppermost three layers and the “light” species defaults for the lower four layers. For all other atoms, the “tight” settings as supplied by FHI-aims were used.

## 3 Results and Discussion

To establish a connection between the surface coverage of TCNE on Cu(111) and the work function, the latter was experimentally determined for a number of samples with different amounts of adsorbed TCNE from the secondary electron cutoff of photoemission spectra (examples are shown in the Supporting Information, Figure S1). Figure 1b shows how the work function changes between the pristine Cu(111) surface (coverage θ = 0) and a surface with the maximum amount of adsorbed TCNE obtainable at room temperature (θ = 1.0). The largest work function change, ΔΦ, of almost +2.9 eV with respect to the clean Cu(111) surface [Φ(θ = 0) = 4.7 eV] is obtained for full monolayer coverage, which corresponds to 3.25 molecules/nm^2^ (see the Supporting Information for coverage determination). In the submonolayer regime, the work function increases monotonously and, despite the substantial scattering of the data, shows only slight deviation from a linear relationship between ΔΦ and TCNE coverage.

To rationalize the result of Figure 1b, we resort to calculated ΔΦ′s for this adsorption system.^27^ For a closed monolayer of TCNE adsorbed exclusively in the face-on geometry (μ4, Figure 1a), being the energetically most favorable adsorption geometry, the calculations predict a minor work function increase of about +0.5 eV due to the counterbalancing effects of electron push-back, charge-transfer dipole, and the negligible perpendicular dipole of the surface-bound nitrile groups. By contrast, in the edge-on geometry (μ2), being less strongly bound, the contributions of electron push-back and charge transfer are reduced, while the dipoles of bonded and free CN groups are not equal anymore (due to local charge transfer involved in the bonding of CN to the Cu surface). This results in a net molecular dipole and a calculated work function increase of up to 3 eV.^27^

From this comparison, it is already clear that an adsorption model with only face-on adsorbed TCNE molecules cannot account for the observed results. On the one hand, the observed maximal coverage of a densely packed face-on (μ4) phase was calculated to be 2 molecules per nm^2^, corresponding to 61% (θ ≈ 0.61 ML in Figure 1b) of the maximal coverage observed experimentally.^19^ On the other hand, the experimentally observed work function increase of approximately 1.5 eV exceeds the theoretical prediction by about a factor of 3. Hence, a realistic scenario has to include upright standing (μ2) molecules, either in short edge-on or long edge-on geometry (Figure 1a), together with μ4 bonded TCNE already at a rather low coverage. A complex binding scenario with TCNE molecules adopting different adsorption geometries was also observed on Co(100).^29^ For this system, distinctly different changes in the work function with coverage were reported. First, the ΔΦ is limited to +1.0 eV. Second, its coverage dependence deviates from the linear correlation observed here. Instead, three regions were identified in which the last one, starting at a coverage of about θ ≈ 0.7 ML, shows an even declining work function with increasing coverage. To understand the cause for the large work function increase observed in the present case and the differences to the Co system, it is necessary to study the interface structure, i.e., the geometric properties of the adsorbed TCNE molecules, in more detail.

**Figure 2:**
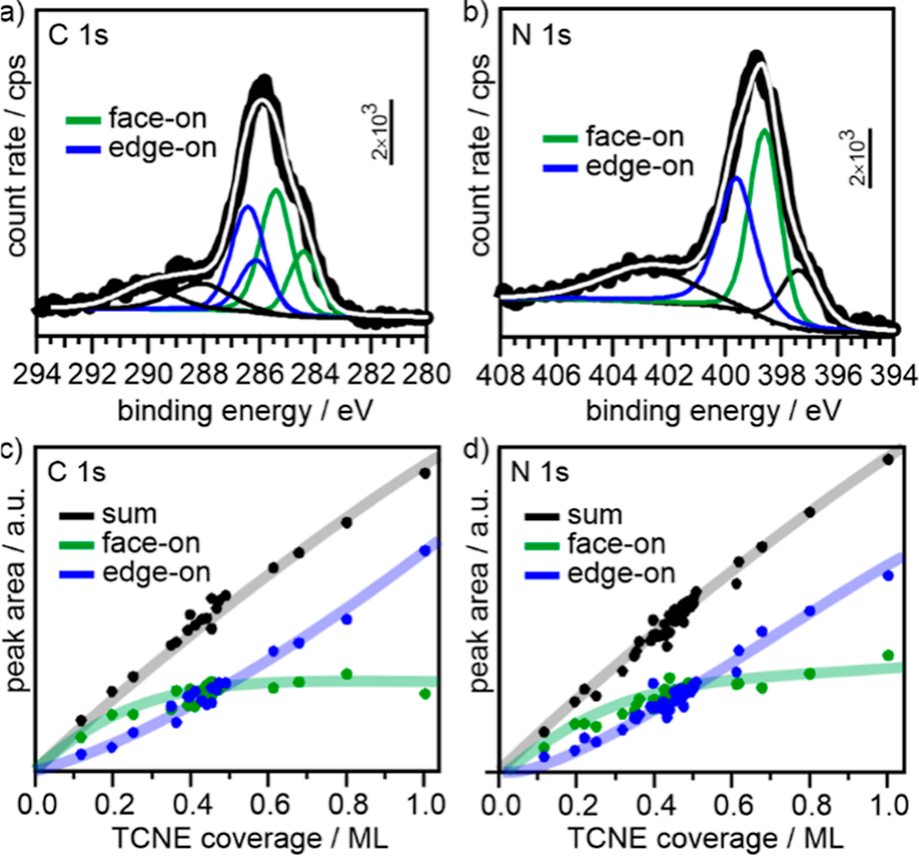
Experimental XP spectra (bold black lines) and fit results (colored lines: individual components of face-on (green) and edge-on (blue) adsorbed molecules; white: fit sum) of the (a) C 1s and (b) N 1s region for 0.5 ML TCNE on Cu(111) (see the Supporting Information for the description of fitting procedure and assignment of components). In parts c and d, the peak area of the individual contributions of face-on (green) and edge-on (blue) molecules, and their sum (black), is plotted as a function of the TCNE coverage.

To this end, we have measured the C 1s and N 1s XP spectra of TCNE on Cu(111) in the (sub)monolayer regime at different coverages. Representative spectra for 0.5 ML coverage are shown in Figure 2a,b, respectively. Although broad and unresolved, a previous study^29^ has shown that the two adsorption geometries exhibit different relative core-level binding energy shifts due to different charge transfer and core-hole screening for edge-on and face-on TCNE. Therefore, the XP signals can be fitted with various components reflecting the different C and N species in the two adsorption geometries (see the Supporting Information for a description of the XP spectra fitting). The results of the analysis are shown in Figure 2c,d, respectively, where the photoemission intensities of the face-on (green) and edge-on (blue) molecules, and their sum (black), are plotted against coverage. A consistent description of the coverage-dependent composition of the adsorbed layers is found for both signals providing evidence for the reliability of the model used for the fitting.

At low coverage, the face-on geometry (green) is dominant, which is expected as this species, according to the theory, exhibits the highest adsorption energy on Cu(111).^30^ However, components of edge-on adsorbed molecules are found already at the lowest coverage analyzed here. The crossing point at which edge-on molecules are predominant is found at about 0.5 ML, which is even below the limiting coverage for a full monolayer of face-on molecules (0.61 ML). Instead, the crossing point is close to the coverage (0.4 ML) at which the intensity of the face-on component saturates. In contrast to that, the amount of edge-on (blue) adsorbed molecules increases slowly at low coverage and then linearly with coverage above about 0.4–0.5 ML, resulting in an almost linear dependence of the total intensity (black) on coverage.

Although the XPS analysis allows to distinguish between face-on and edge-on adsorbed TCNE molecules, it is not able to provide information on what type of edge-on species, i.e., long edge-on or short edge-on (Figure 1a), are present on the surface. In this regard, vibrational spectroscopy can provide further insights. In previous HREELS experiments, multiple bands were observed for TCNE on Cu(111) in the monolayer regime, which were assigned to differently charged TCNE species.^21^ In a subsequent IRRAS study, the HREELS results were confirmed.^22^ However, the number of observed vibrational modes in IRRAS was much lower because the metal surface selection rule, which governs the intensity of dipole transitions in IRRAS, limits the observable vibrational modes to those that have a transition dipole moment component perpendicular to the surface,^37^ a condition which does not apply to impact scattering in HREELS. Nevertheless, IRRAS can potentially be used here in combination with the results of XPS to distinguish between long edge-on and short edge-on TCNE molecules. This is because the ν(C=C) symmetric stretching vibration of TCNE, which is only Raman active for molecules with a *D*_2*h*_ symmetry, becomes IR-active upon adsorption on a surface. Although the *D*_2*h*_ symmetry is broken in all adsorbed states of the molecule, the metal surface selection rule will restrict the observation of the ν(C=C) mode to the short edge-on geometry, as the transition dipole moment of this mode is considered parallel to the C=C bond and thus parallel to the surface for all other μ4 and μ2 adsorption geometries.

**Figure 3:**
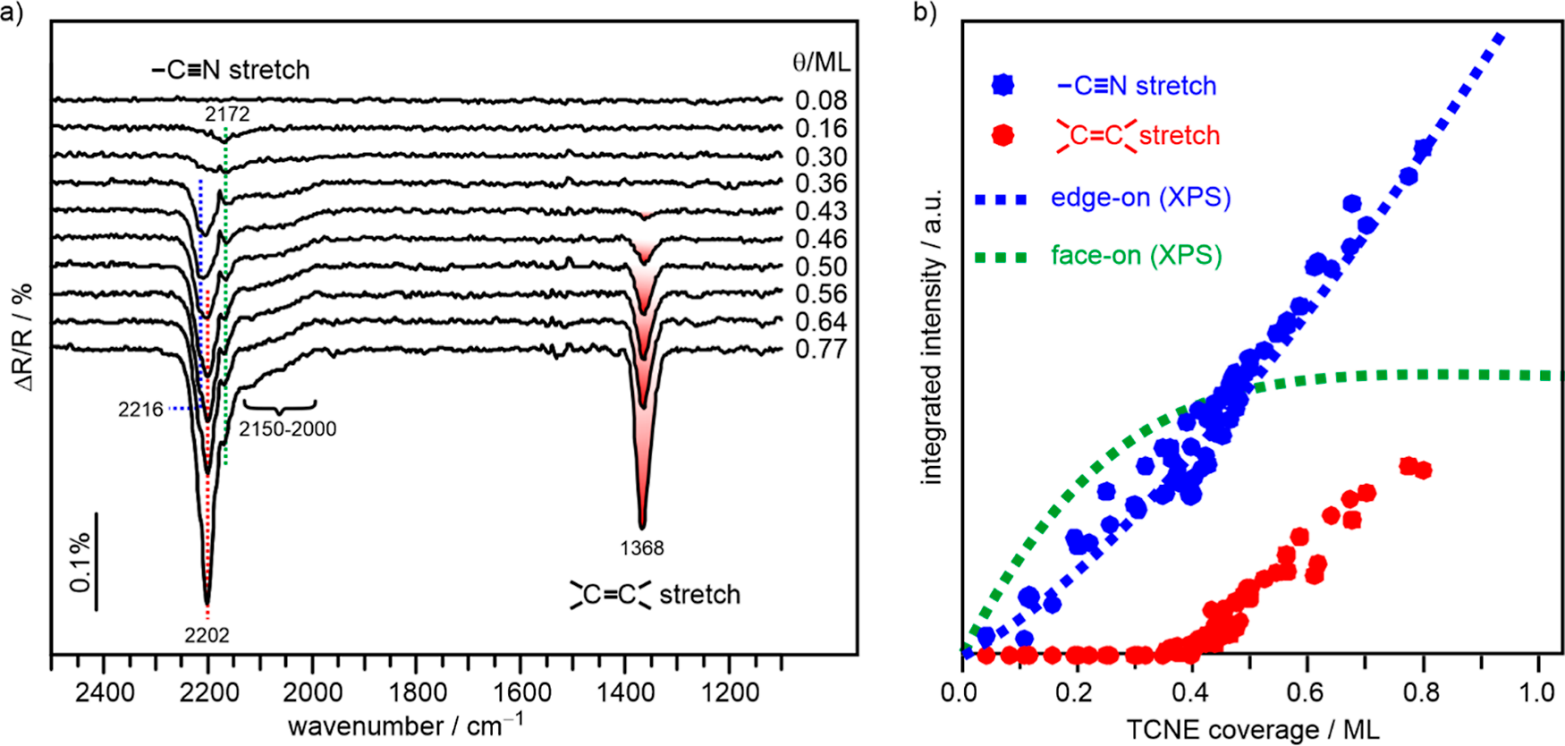
(a) IRRA spectra of TCNE/Cu(111) at different coverages. (b) Intensities of the signals in the range of the CN stretching vibrations (2000–2220 cm^–1^) (blue symbols) and the symmetric C=C stretching vibration (1370 cm^–1^) (red symbols) as a function of coverage. For comparison, the curves for the XPS-derived intensities of the edge-on (dotted blue line) and the face-on (dotted green line) adsorbed molecules from Figure 2 are also included in this plot.

Figure 3a shows the IRRA spectra of TCNE/Cu(111) for increasing coverage in the (sub)monolayer regime. At high coverage, two main features characterize the spectra. One around 2200 cm^–1^ can be assigned to ν(CN) stretching vibrations, and the other at 1368 cm^–1^ is related to the symmetric ν(C=C) stretching vibration. At low TCNE coverage (0.16 ML), the first signal that appears is a CN stretching vibration at 2170 cm^–1^, which remains unchanged with further TCNE dosing. However, additional CN bands grow in at increasing coverages: broad unresolved contributions in the range of 2150–2000 cm^–1^ and more distinct features between 2175 and 2220 cm^–1^. We note that up to 0.4 ML no additional vibrational signals except of the CN stretching vibrations are observed. Above a coverage of >0.4 ML, an additional signal is observed at 1360 cm^–1^, which increases in intensity with coverage and shifts to 1368 cm^–1^ when reaching saturation. This signal can be assigned to an ν(C=C) vibration and is accompanied by a CN vibration at 2202 cm^–1^, which grows with increasing coverage together with the ν(C=C) vibration and becomes the dominating feature in the ν(CN) region at high TCNE coverages.

The assignment of the observed vibrations requires knowledge of the adsorption geometries and charge-transfer characteristics. For bulk (solid) TCNE, two of the four CN stretching vibrations are IR active and found at 2260 and 2220 cm^–1^.^38^ Upon adsorption on the surface, the CN bond strengths, and therefore the stretching frequency, are reduced due to the local bond formed between the Cu surface atoms and the N atoms of the CN groups, and the associated local charge transfer.^39^ This is consistent with our observations and with previous results.^21,22^ Additionally, charge transfer from the metal into the molecules’ LUMO upon adsorption can lead to further reduction of the CN stretching frequency.^39^ We thus assign signals below 2180 cm^–1^ to vibrations of bonded CN groups and those above to free CN groups. Concerning the ν(C=C) vibrations, in bulk TCNE the corresponding Raman band is at 1573 cm^–1^.^38^ In our and previous IRRAS investigations,^22^ the ν(C=C) was found at 1368 cm^–1^, which is consistent with a charge transfer to the molecule leading to a weakening of the double bond and thus to a red shift of the vibrational band. In addition, as the transition dipole moment of this band is aligned with the C=C bond, the metal-surface selection rule suggests that this bond exhibits a significant angle with the surface as found, e.g., in the short-edge-on adsorption geometry. Importantly, this C=C vibration appears only at TCNE coverages >0.4 ML, which allows the edge-on contribution of the XPS signal to be decomposed with respect to the different adsorption geometries. For this, we plot the integrated IR intensities of the CN (blue symbols) and C=C vibrations (red symbols) as a function of TCNE coverage (Figure 3b). Because the CN bonds of face-on adsorbed TCNE are oriented almost parallel to the surface and, therefore, do not contribute significantly to the signal, we assume that the CN signal intensity is mainly from μ2 adsorbates with either short edge-on or long edge-on adsorption geometry. This is consistent with the lack of an IR band in the CN range for very low coverage, where it is assumed that a large proportion of the molecules is adsorbed in a face-on geometry. Correspondingly, the coverage-dependent evolution of the CN IR signal intensity should correlate with the XPS intensity of edge-on species, which is indeed the case, as shown in Figure 3b by the blue dotted line representing the corresponding XPS intensity (taken from Figure 2c and scaled to fit the IR intensity). The complete absence of an ν(C=C) IR signal between 0 and 0.4 ML coverage clearly indicates that in this coverage range the predominant edge-on species on the Cu(111) surface is the long edge-on species. Only at coverages >0.4 ML, the short edge-on species, which gives rise to the ν(C=C) signal at 1368 cm^–1^, are found.

The dosage-dependent evolution of CN and C=C signals observed here for TCNE adsorption on Cu(111) at room temperature is generally similar as in earlier IRRAS observations for adsorption at 100 K.^22^ In the earlier work, the characteristic C=C IR signal at 1368 cm^–1^ was assigned to singly charged TCNE in the second layer, while the first layer was assumed to be composed of only doubly charged TCNE with a face-on adsorption geometry. Experiments at low adsorption temperatures are complicated by the formation of TCNE multilayer islands already at submonolayer coverage. Because second-layer formation can safely be excluded under the experimental conditions employed here (see the Supporting Information), a realistic model of the adsorption structure of TCNE on Cu(111) in the monolayer regime has to include a distribution of various TCNE adsorption geometries, as shown in Figure 1a, which is consistent with the conclusions of more recent experimental and computational studies.^19,29^

**Figure 4:**
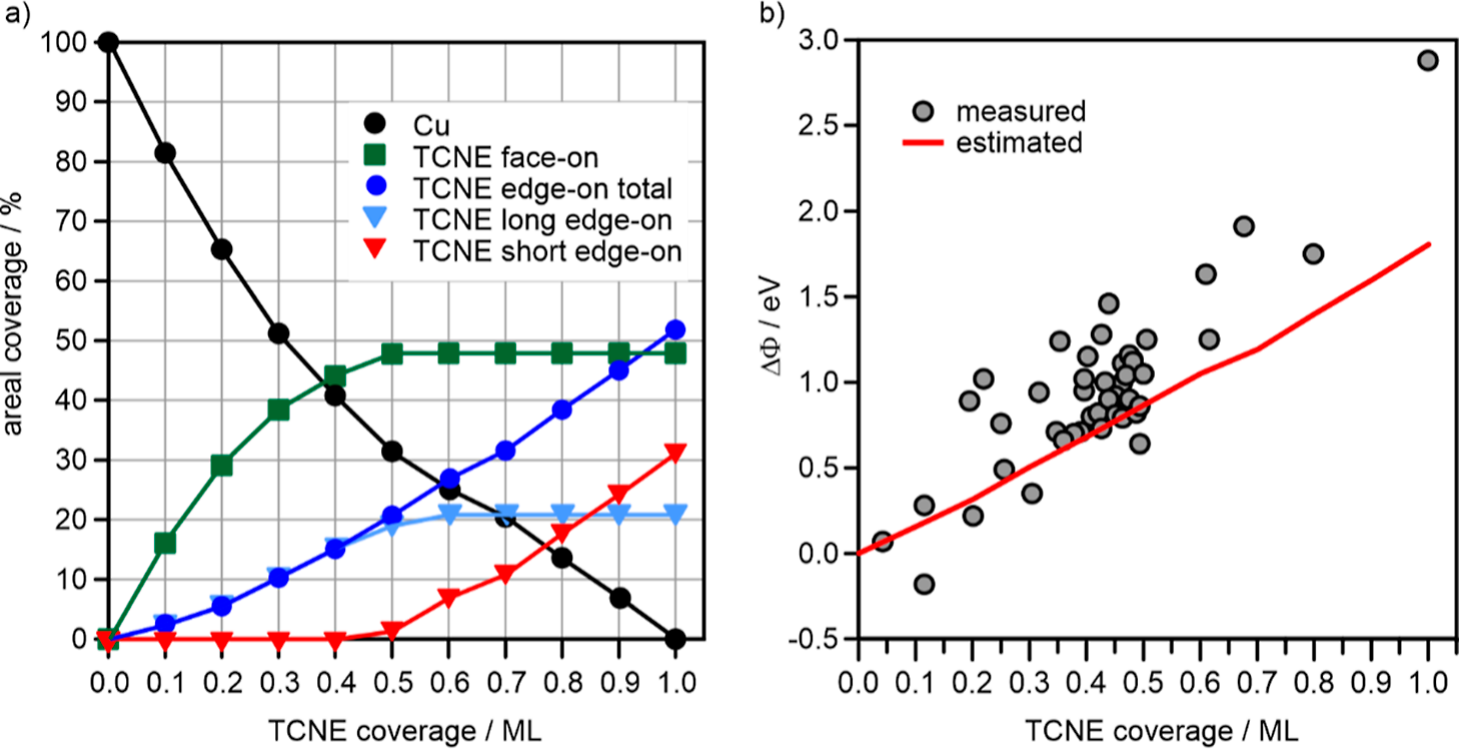
(a) Plot of the areal coverage of various TCNE species and of free Cu surface as a function of total TCNE coverage. (b) Comparison of the experimentally measured work function change (ΔΦ) as a function of TCNE coverage (points) to the one estimated from the areal distribution of different species (red line).

The information gained from XPS and IRRAS can finally be combined to draw a quantitative picture of the distribution of the different TCNE species in the monolayer. In Figure 4a, we plot the areal coverage of the identified TCNE species as a function of TCNE coverage. To calculate the areal coverage, we refer to computational data, which determined the densest monolayer structures of TCNE in the face-on and edge-on configurations to have a density of 2.0 and 4.4 molecules/nm^2^, respectively.^19^ Our XPS measurements of the TCNE/Cu(111) interface in the (sub)monolayer regime show two different classes of TCNE, which we identified as face-on and edge-on orientations by comparison with previous experiments and first-principles calculations.^22,29^ As predicted by these calculations, the face-on geometry (μ4) dominates at low coverages due to its higher adsorption energy at the single molecule level compared to the other geometries. However, experimentally, the edge-on geometry (μ2) is also present at low coverages. A possible explanation for this behavior is the formation of highly disordered islands due to the attractive interactions between the TCNE molecules,^19^ which allow for trapping of additional TCNE molecules preferably in an edge-on orientation in the case of limited available open Cu sites.

An indication of structural disorder of the adsorbed layer is the complete lack of a LEED pattern after TCNE adsorption. Furthermore, STM measurements of the TCNE/Cu(111) interface show that individual TCNE molecules become mobile already at 55 K.^40^ This interpretation is further corroborated by our observations with increasing coverage. Adsorption of TCNE with the face-on geometry saturates at 0.4 ML, while the edge-on species continue to adsorb and eventually become dominant at the interface. This is because at 0.4 ML the surface is covered by a highly disordered layer with few available surface sites for adsorption in the face-on geometry, only allowing further adsorption in the edge-on orientation. Another explanation for the stabilization of the edge-on species at a higher coverage is provided by the increased stability of TCNE adsorbates with a smaller footprint; i.e., the adsorption energy per area is larger for edge-on than for face-on adsorption. In addition, the 1367 cm^–1^ ν(C=C) band appears in the IRRA spectra at 0.4 ML coverage, indicating the emergence of the second edge-on species, whose C=C bond is oriented perpendicular to the surface (TCNE short edge-on, Figure 4a). We assume this species to experience a similar degree of charge transfer as the other edge-on species and therefore to be practically indistinguishable from it in XPS.

With the TCNE areal coverage derived from the spectroscopic data (Figure 4a), we finally estimate the work function change and compare it to the measured ΔΦ. For the ΔΦ of the different adsorption geometries we again refer to computational studies, which suggest a maximum induced ΔΦ of +0.5 eV for the face-on geometry and of +3 eV for the edge-on geometry.^19,27^ Combining this with the results of the present study gives an estimated ΔΦ as a function of TCNE coverage shown by the red line in Figure 4b. Compared to the experimental data, the model underestimates the ΔΦ by about 30%, but otherwise reproduces the trend of an almost linear work function increase with coverage well. Due to the large difference in the induced ΔΦ of face-on and edge-on TCNE molecules, it is assumed that the work function change is mainly due to the large contribution of edge-on molecules. Indeed, ΔΦ follows the trend of the areal coverage of those molecules (Figure 4a, TCNE edge-on total).

**Figure 5:**
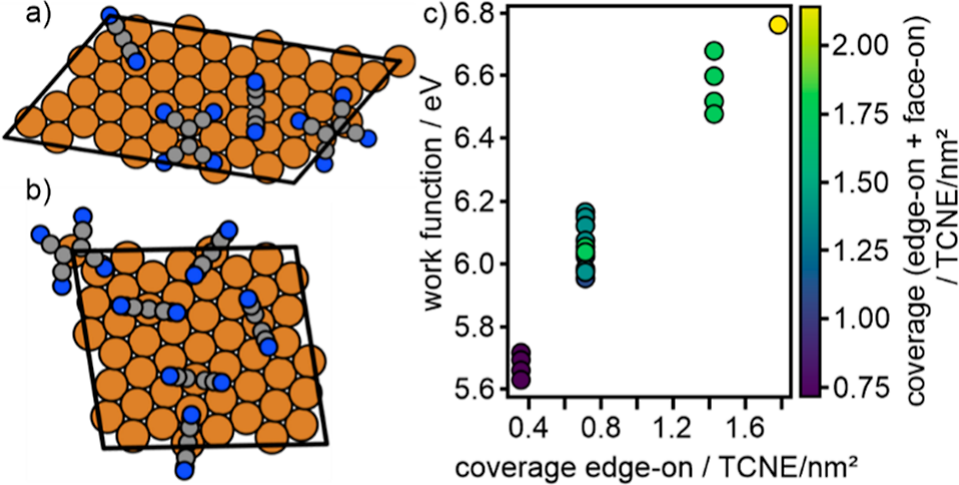
Examples of models for TCNE structures with (a) 4 TCNE molecules (2 face-on and 2 edge-on) and (b) 6 TCNE molecules (1 face-on and 5 edge-on) used to calculate the work function of mixed structures and its dependence of the number of edge-on and face-on molecules. (b) Calculated work function as a function of the coverage of edge-on molecules in the various structures. The color corresponds to the total coverage shown in the bar on the right.

To support this assertion, we computationally analyzed the ΔΦ of mixed TCNE structures, as suggested by the experimental data. For this purpose, several structures representing different coverages and face-on/edge-on ratios were randomly generated in a building block approach using the adsorption geometries determined in an earlier work.^19^ Both kinds of edge-on adsorption geometries were used (long edge-on and short edge-on) in various ratios to the coverage of face-on molecules. In Figure 5, two examples of mixed structures, one with 2 face-on and 2 edge-on molecules (Figure 5a, 1.42 TCNE/nm^2^), and one with 1 face-on and 5 edge-on molecules (Figure 5b, 2.12 TCNE/nm^2^), are shown. The plot of the work function of these structures as a function of the coverage of edge-on TCNE molecules (Figure 5c) shows an almost linear correlation between ΔΦ and the coverage of edge-on bound TCNE with a Pearson correlation coefficient of 0.97, confirming the qualitative model discussed above. Furthermore, when comparing the work function of the reference structures with that of the structures where the face-on molecules had been removed, we find that the work function remains virtually unchanged (see the Supporting Information), which means that the coverage of face-on molecules does not significantly contribute to the work function of the Cu(111)/TCNE monolayer system.

This result further demonstrates that the main contribution to the interface work function in the TCNE/Cu(111) system is the dipole of the free nitrile groups of the edge-on TCNE molecules, while the dipoles of the bonded nitrile groups and the ones induced by electron push-back and charge transfer, which could have significant values individually, cancel each other out. In the case of a high coverage of negatively charged molecules, depolarization effects are to be expected, which tend to decrease the work function in an adsorbate/substrate system. This effect was used to explain the declining work function at high coverage for TCNE adsorption on Co(100).^29^ For TCNE on Cu(111) in the monolayer regime, this effect does not contribute significantly because of the cancellation effects mentioned above and possibly a different ratio and distribution of edge-on/face-on molecules compared to Co(100), resulting in a monotonous increase of the work function until saturation of the monolayer.

## 4 Conclusions

In this study, we have experimentally investigated the formation of a TCNE monolayer on Cu(111) using work function measurements, XPS and IRRAS. The TCNE molecules adsorb in different geometries depending on the coverage. In addition to molecules with a face-on adsorption geometry (flat lying), which initially dominate, edge-on molecules (upright standing) are also trapped at low coverages. Once the number of face-on molecules saturates at around half of the monolayer coverage, edge-on molecules become the dominating species at high coverage. With XPS and IRRAS it was not only possible to distinguish between face-on and edge-on molecules but also to discriminate between different edge-on (short edge-on and long edge-on) species and to finally draw a quantitative picture of the areal distribution of different species depending on the coverage. Our results suggest that the monolayer, rather than being composed of single phases of face-on or edge-on molecules, consists of mixed structures, which occur due to the interplay of small barriers for molecular diffusion and transition between flat and upright structures and the availability of appropriate adsorption sites for molecules in the different adsorption geometries. As the monolayer was only observed for few hours, there are no indications on how long these mixed structures prevail and if they transform into ordered phases, as predicted in an earlier work.^30^

The increase in the Cu(111) work function with increasing TCNE coverage shows an almost linear trend and no sign of depolarization at high coverages. Combined with the spectroscopic results, this trend can be explained by a direct relation between the work function increase and the number of molecules with an edge-on geometry in the monolayer and, correspondingly, the dipole of free nitrile groups. Our study thus provides a link between an easily accessible macroscopic system property (work function) and the microscopic details given by the distribution of molecules with different geometries at the interface. The maximum work function change obtained at full monolayer coverage is 2.9 eV, which gives an absolute work function of 7.6 eV for the TCNE/Cu(111) system. Because our results suggest the formation of mixed rather than phase-separated structures and because of the small size of the TCNE molecules, we do not expect variations in the local work function across the surface.

## Supporting Information Available

Secondary electron cutoff spectra, monolayer coverage determination, XP spectra fitting, and additional computational results (PDF)
