# Supplementary material for: Interplay of Adsorption Geometry and Work Function Evolution at the TCNE/Cu(111) Interface
Source: J Phys Chem C Nanomater Interfaces. 2023 Dec 8;127(50):24266–73. doi: 10.1021/acs.jpcc.3c06422 (PMC10749461; doi:10.1021/acs.jpcc.3c06422)
Supplement: Supplementary file 1 — jp3c06422_si_001.pdf [file jp3c06422_si_001.pdf]

Supporting Information

Interplay of Adsorption Geometry and Work Function Evolution at the TCNE/Cu(111) Interface

Max Niederreiter,[1] Johannes Cartus,[2] Anna Werkovits,[2] Oliver T. Hofmann,[2] Thomas Risse,[3] Martin Sterrer[1]

[1] Institute of Physics, University of Graz, NAWI Graz, Universitätsplatz 5, 8010 Graz, Austria.

[2] Institute of Solid State Physics, Graz University of Technology, NAWI Graz, Petersgasse, 16/II, 8010 Graz, Austria.

[3] Institute of Chemistry and Biochemistry, Freie Universität Berlin, Arminallee 22, 14195 Berlin, Germany.

SI1) Secondary electron cut-off

Figure S1 shows secondary electron cut-off spectra of selected samples (clean Cu(111), and Cu(111) with 0.4 ML and 1 ML adsorbed TCNE, respectively). The spectra were obtained with the XPS set-up and with a bias voltage of ~100 V applied to the sample (the exact applied bias was obtained for each sample separately by determining the shift of the Cu $2p_{3/2}$ peak position of the biased sample with respect to the Cu $2p_{3/2}$ peak position of the non-biased (grounded) sample).

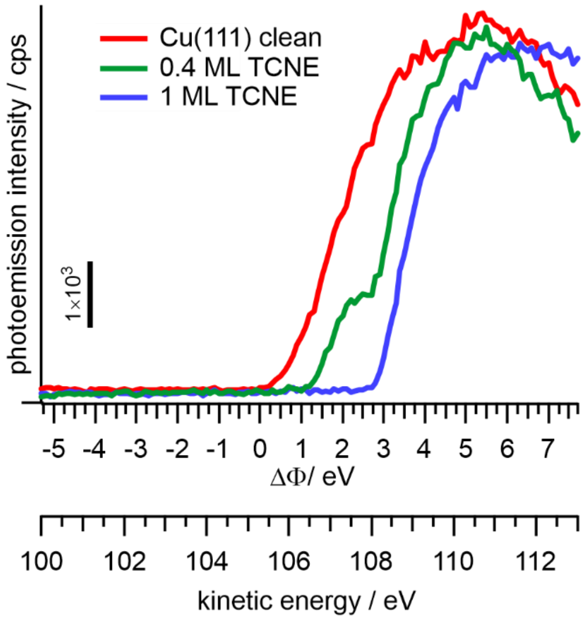

Figure S1. Secondary electron cut-off spectra of clean Cu(111) (red), and Cu(111) with two different coverages of TCNE (green: 0.4 ML TCNE; blue: 1 ML TCNE). The spectra are displayed on the kinetic energy scale and the $\Delta\Phi$ scale (where $\Delta\Phi$ is the work function change of Cu(111) upon adsorption of TCNE), respectively. The spectra were recorded in a sample bias configuration with an applied bias of ~100 V.

SI2) Monolayer coverage

To achieve different coverages in the monolayer regime, TCNE was dosed on Cu(111) from the gas-phase in two different ways: either at room temperature at a specific background dosing pressure (up to 5×10^{-7} mbar) of TCNE for a specific time or until saturation at the chosen conditions was reached; or at 200 K until a multilayer of TCNE was formed, followed by warming up to room temperature. The highest monolayer coverage, as determined with XPS, was achieved by the latter procedure, and is referred to as one monolayer (1 ML) in this work. Figure S2 shows the evolution of the C 1s, N 1s, and Cu 2p XP signals of the initially grown TCNE multilayer upon warming the sample from 200 K to 300 K. After a decrease of C 1s and N 1s XP intensities around 235 K assigned to the desorption of the multilayer,[1] a stable intensity of both XP signals was observed up to 300 K. The latter establishes a stable TCNE monolayer in this temperature window and rules out the formation of a stable second layer above the monolayer observed for several small organic adsorbates, because these layers are generally found to be only slightly more stable than the multilayer.[2, 3]

To further quantify the monolayer coverage, we refer (i) to Figure 2 in the main manuscript, which provides the molecular distribution of face-on and edge-on adsorbed molecules (30:70) for 1 ML coverage from XPS analysis, and (ii) to previous computational data,[4] which determined the densest monolayer structures of face-on and edge-on phases to exhibit densities of 2.0 and 4.4 molecules/nm^2, respectively. The areal distribution of face-on and edge-on molecules is then calculated to be 0.48:0.52. From this, the molecular density within the full monolayer of TCNE on Cu(111) is calculated to be approximately 3.25 nm^{-2}. This value is only slightly higher than the TCNE saturation molecular density on Co(100) (3.2 nm^{-2}).[5]

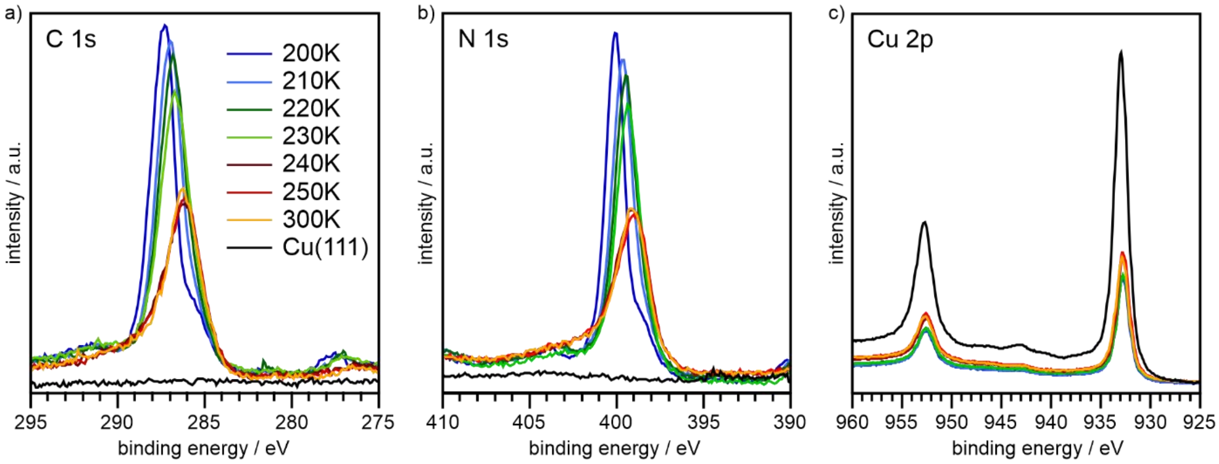

Figure S2. XP spectra of the C 1s (a), N 1s (b), Cu 2p (c) regions of clean Cu(111) (black), a Cu(111) surface covered with a TCNE multilayer at 200 K, which was subsequently heated stepwise to the temperatures given in the legend in (a). The TCNE multilayer desorbs between 230 K und 240 K, leaving a TCNE monolayer on the surface.

SI3) XP spectra fitting and interpretation

From the combination of work function measurements and computational results it was concluded that differently oriented TCNE species should be present already at rather low coverage on Cu(111) (see main manuscript). In a previous work it was shown that information about the distribution of face-on and edge-on molecules on the surface is contained in the C 1s and N 1s XP spectra of adsorbed TCNE, as differences in charge transfer and core-hole screening give rise to different relative core-level binding energy shifts for the two species.[5] The spectra fitting routine and explanations of binding energy shifts are very well described and documented in Ref. [5] and are summarized below for the present case.

Fig. S3 redisplays the XP spectrum of the N 1s and C 1s region at 0.5 ML coverage shown also in the main manuscript (Figure 2). The N 1s signal (Fig. S3a) consists of a main peak centered around 399 eV binding energy and a shake-up satellite at about 403 eV.[6] The main peak possesses a small, low binding energy shoulder, which is attributed to defect adsorption and to TCNE fragments that have been reported in literature.[7] The main N 1s peak is broad and consists of several unresolved components from the face-on and edge-on TCNE molecules. For the face-on species, where all 4 N atoms of the nitrile groups are bonded to the surface, we expect a single N 1s peak, which should be located at the low binding energy side of the signal due to the enhanced charge transfer into the face-on species and the more effective core-hole screening. For TCNE molecules in edge-on geometry two different N 1s signals arising from surface bound and “free" nitrile groups, respectively, are expected. Due to the reduced charge transfer predicted by theory,[4] their signals should be located at the high binding energy side of the signal from the face-on molecules. However, the spectral resolution is insufficient to separate the individual contributions. The presence of two components is therefore accounted for by using a larger line width for the signal of these species in the spectral fitting.

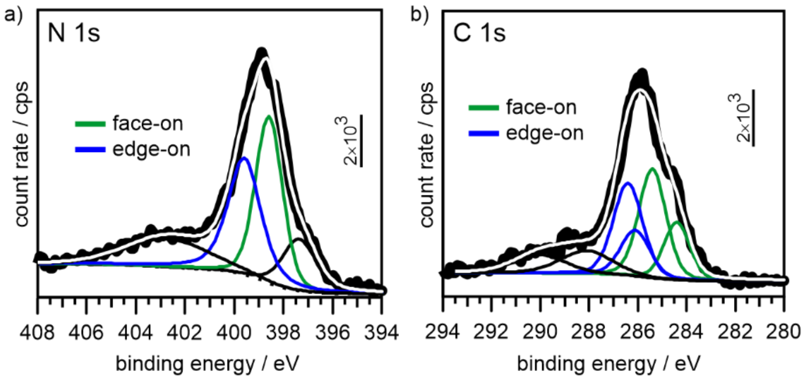

Figure S3. Experimental XP spectra (bold black lines) and fit results of the a) N 1s and b) C 1s region for 0.5 ML TCNE on Cu(111).

The corresponding C 1s XP spectrum (Figure S3b) displays a similar structure as the N 1s spectrum. TCNE possesses two inequivalent classes of carbon atoms previously shown to exhibit different binding energy.[6] The two carbon atoms of the central vinyl group belong to one and the four of the peripheral nitrile groups to the other class. For a delineation of the XP spectra each orientation of the TCNE molecules requires two peaks with a fixed relative intensity ratio of 2:1 according to the atomic ratio of CN and CC bonded carbon in the molecule. Note that the splitting between the lines is different for the edge-on and face-on species.[5] As in the case of the N 1s signal, the contributions of face-on and edge-on species are subject to different core-level binding energy shifts due to charge-transfer and core-hole screening effects, locating the face-on contributions at lower, and the edge-on contributions at higher binding energy. In addition to the four C 1s lines, two shake-up satellites for the main peak of the two orientations are fitted into the broad tail on the high binding energy side.

SI4) Work function of mixed structures – computational results

In Figure 5 in the main manuscript it is shown that the work function correlates with the coverage of edge-on adsorbed TCNE molecules on Cu(111). From this, it was concluded that mainly this class of molecules contributes to the interface dipole. To further substantiate this conclusion, we provide in Figure S4 the analysis of the contribution of face-on adsorbed molecules to the work function for the same mixed structures with different coverages and edge-on/face-on distribution. In these structures no clear trend is seen, and the Pearson correlation coefficient between the coverage of face-on molecules and the work function is roughly zero, suggesting that the two quantities are uncorrelated.

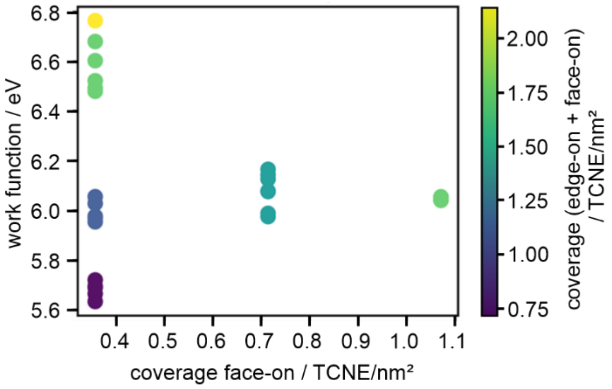

Figure S4. Calculated work function as a function of the coverage of face-on molecules in various randomly generated mixed (face-on and edge-on) structures. The color corresponds to the total coverage shown in the bar on the right.

In addition, we investigated how the work function of mixed structures is affected when the face-on molecules are removed. Again, the same randomly generated mixed structures were used. Figure S5 shows one example of the original structure (Figure S5a) and the one with the face-on molecules removed (Figure S5b). When comparing the work function (Φ) of the structures where the face-on molecules were removed (edge-on only) to that of the original structures (edge-on + face-on), we find that the work function is almost identical (Figure S5c). This shows again that the impact of the face-on molecules on the surface dipole is small.

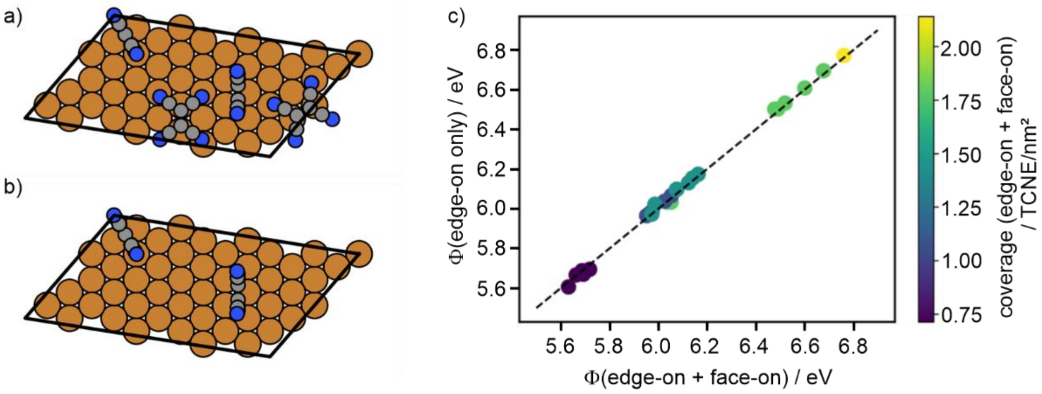

Figure S5. a) Mixed structure consisting of two face-on and two edge-on TCNE molecules on Cu(111). b) same as in a) with face-on TCNE removed. c) Plot showing the correlation of work functions (Φ) of the mixed (face-on and edge-on) structures and the ones where the face-on molecules have been removed. The color corresponds to the total coverage shown in the bar on the right.

References

(1) Pan, F. M.; Hemminger, J. C.; Ushioda, S., Adsorption of tetracyanoethylene on a Ni(111) surface studied by Auger electron spectroscopy, thermal desorption spectroscopy, and Raman spectroscopy. *J. Phys. Chem.* **1985,** 89, 862-867.
(2) Ould Hamou, C. A.; Réocreux, R.; Sautet, P.; Michel, C.; Giorgi, J. B., Adsorption and decomposition of a lignin β-O-4 linkage model, 2-phenoxyethanol, on Pt(111): Combination of experiments and first-principles calculations. *J. Phys. Chem. C* **2017,** 121, 9889-9900.
(3) Réocreux, R.; Ould Hamou, C. A.; Michel, C.; Giorgi, J. B.; Sautet, P., Decomposition mechanism of anisole on Pt(111): Combining single-crystal experiments and first-principles calculations. *ACS Catal.* **2016,** 6, 8166-8178.
(4) Egger, A. T.; Hörmann, L.; Jeindl, A.; Scherbela, M.; Obersteiner, V.; Todorović, M.; Rinke, P.; Hofmann, O. T., Charge transfer into organic thin films: A deeper insight through machine-learning-assisted structure search. *Adv. Sci.* **2020,** 7, 2000992.
(5) Lach, S.; Altenhof, A.; Shi, S.; Fahlman, M.; Ziegler, C., Electronic and magnetic properties of a ferromagnetic cobalt surface by adsorbing ultrathin films of tetracyanoethylene. *Phys. Chem. Chem. Phys.* **2019,** 21, 15833-15844.
(6) Capitán, M. J.; Álvarez, J.; Navio, C., Study of the electronic structure of electron accepting cyano-films: TCNQversusTCNE. *Phys. Chem. Chem. Phys.* **2018,** 20, 10450-10459.
(7) Erley, W.; Ibach, H., Spectroscopic evidence for surface anion radical formation of tetracyanoethylene adsorbed on Cu(111) at 100 K: a high-resolution electron energy loss study. *J. Phys. Chem.* **1987,** 91, 2947-2950.
